# A case of mild‐to‐intermediate left‐main lesion with high‐risk plaque features: “Blindness of physiology” for PCI guidance?

**DOI:** 10.1002/ccr3.3197

**Published:** 2020-09-02

**Authors:** Arif Al‐Nooryani, Wael Aboushokka, Zlatko Mehmedbegovic, Branko Beleslin

**Affiliations:** ^1^ Al Qassimi Hospital Sharjah UAE; ^2^ Clinic for Cardiology Clinical Center of Serbia Belgrade Serbia; ^3^ Medical Faculty University of Belgrade Belgrade Serbia

**Keywords:** fractional flow reserve, intermediate left‐main stenosis, optical coherence tomography, unstable plaque

## Abstract

In patients presenting with acute coronary syndrome without ST elevation, both FFR and OCT imaging may be necessary to adequately interrogate patients with intermediate and ambiguous left‐main coronary stenosis.

## Graphical abstract

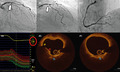

## 1 INTRODUCTION

A 73‐year‐old patient presented with unstable angina and intermediate left‐main coronary stenosis. Fractional flow reserve demonstrated nonsignificant lesion, whereas optical coherence tomography revealed multiple imaging phenomena consistent with plaque instability which over‐ruled initial decision not to intervene, and the lesion was treated with stenting.

*The Beatles song “Let it be” is one of the most universally beloved, remastered and reproduced songs of all time during its half‐a‐century existence since 1970 when it was first aired. Among many, Ray Charles interpretation of the hit, besides being ultimate hearing experience, adds to it a strong personal profoundness and perfect prologue to clinical scenario ahead*.

The pivotal role of fractional flow reserve (FFR) to guide coronary revascularization in patients with stable angina is well defined and included in the guidelines ^1^, ^2^; however, its accuracy is uncertain and not adequately investigated in patients with acute coronary syndrome (ACS) without ST elevation.^3^ In fact, it has been shown that vasodilator microvascular capacity is maintained and similar in patients with non‐STEMI and stable angina ^4^ which enables reliable hemodynamic measurement of FFR. However, patients with ACS may have underlying and by angiography alone nonvisible plaque rupture or erosions, which provide additional and prognostically important information to guide coronary intervention.^5^

Optimal diagnostic strategy for the treatment of LM in patients presenting with acute coronary syndrome without ST elevation remains challenging in the setting of mild‐to‐intermediate and ambiguous angiographic findings, as no randomized studies have been performed to guide practical decisions.

## 2 CASE PRESENTATION

**Figure 1:**
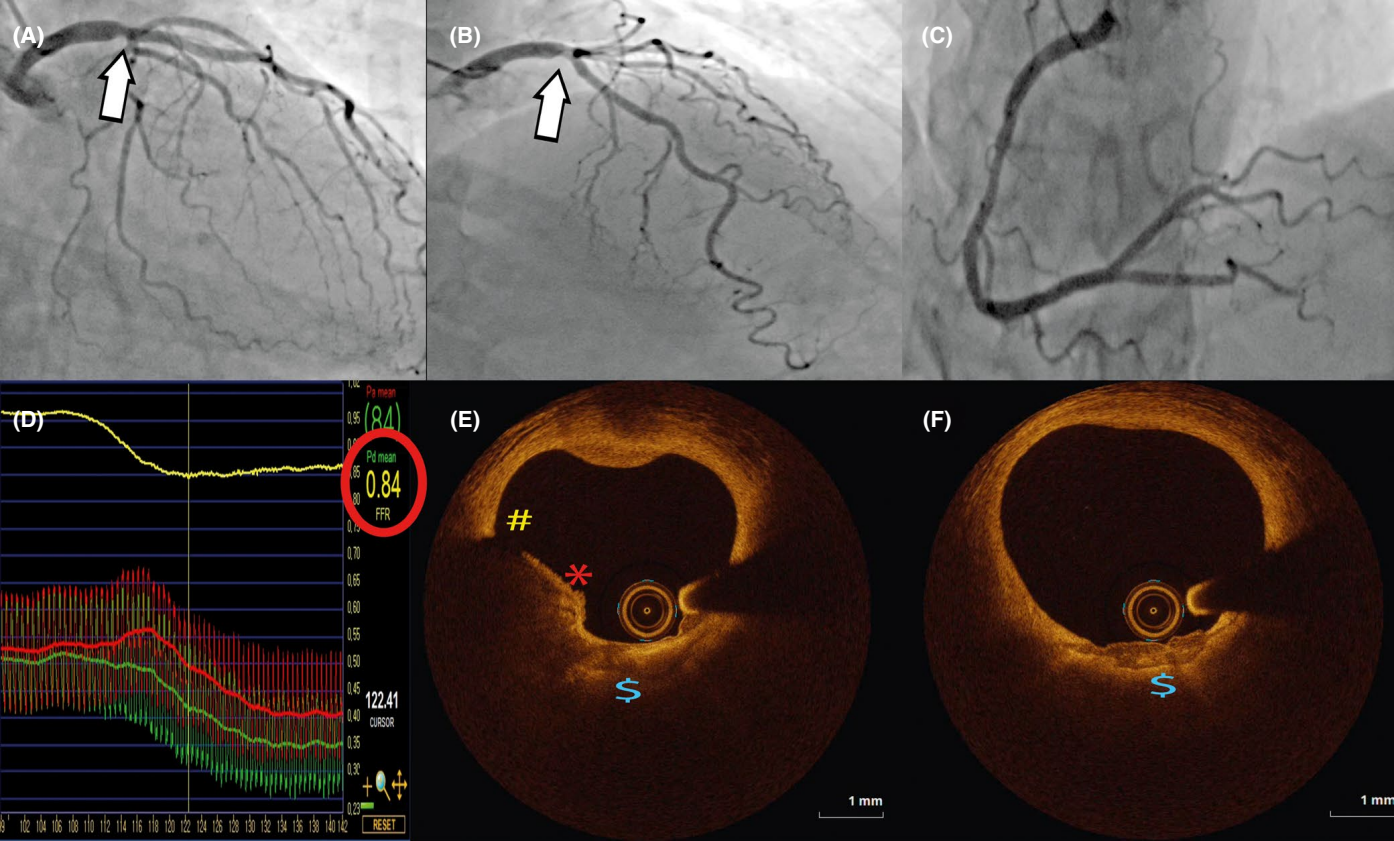
Angiographic, hemodynamic, and imaging evaluation of distal left‐main lesion. Panels A, B, and C: intermediate lesion on distal left‐main (white arrow) and mild disease in right coronary artery; panel D: fractional flow reserve with pressure wire positioned in circumflex coronary artery during maximal hyperemia 0.84; panels E and F: OCT findings at the distal left‐main site—plaque rupture (yellow number sign), plaque erosions with border thrombi (red asterisk), large lipid pool with thin fibrous cap and clusters of macrophages (blue dollar sign). OCT—optical coherence tomography

**Figure 2:**
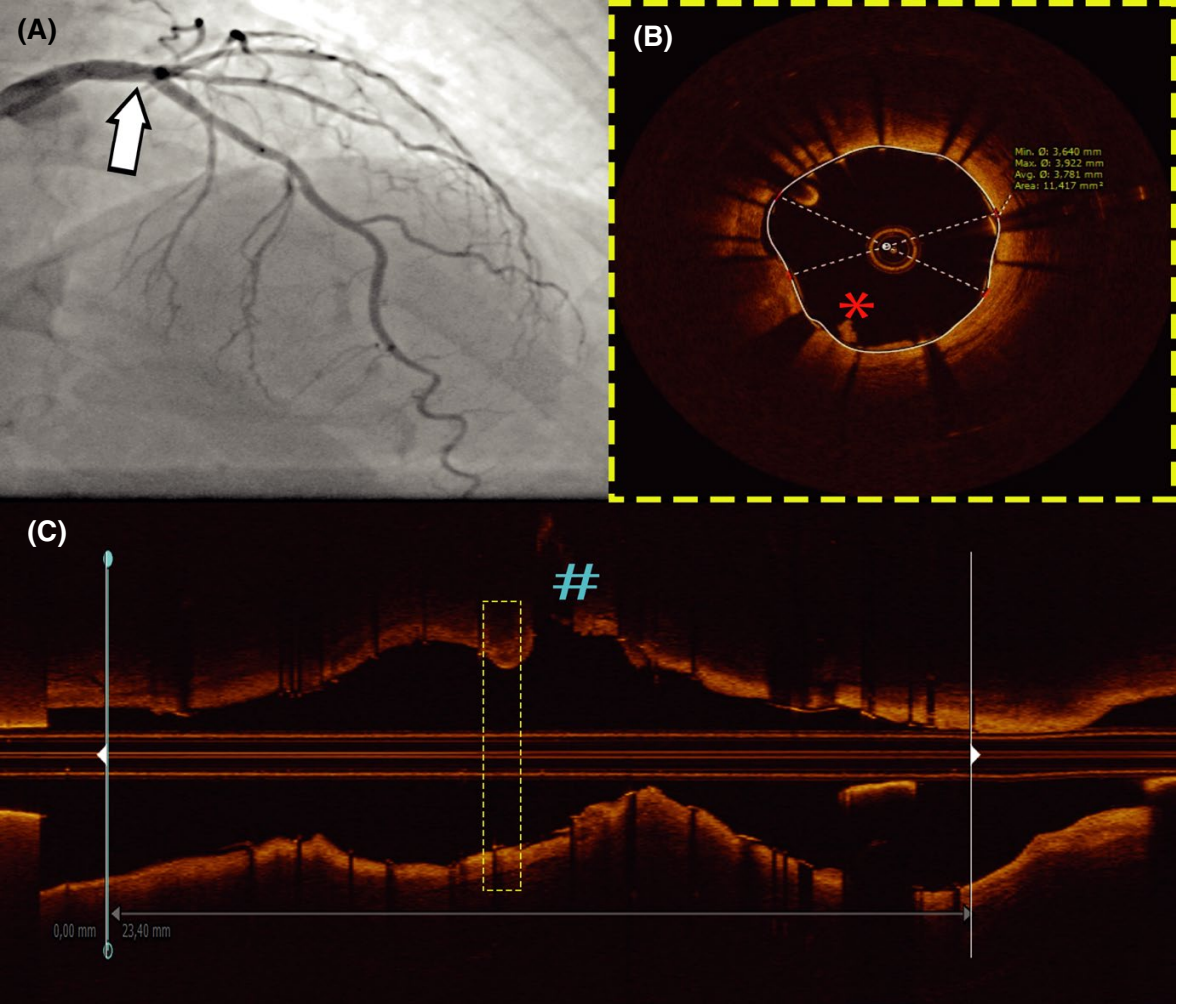
Post‐PCI angiographic and imaging result. Panel A: angiographic result following stenting (white arrow); panel B: OCT findings at the distal left‐main site with good minimal stent area 11.5 mm^2^ showing residual white thrombi prolapse (red asterisk); panel C: longitudinal OCT pullback showing good apposition and fractal bifurcation anatomy reconstruction with ostium of circumflex depicted with blue number sign. OCT—optical coherence tomography; PCI—percutaneous coronary intervention

We present a case of 73‐year‐old female patient with de novo, crescendo angina on effort with some short episodes of resting chest pain in the last 10 days. On admission, her ECG was normal and without ST segment changes. Her cardiovascular risk profile included hyperlipidemia (treated with statins) and positive family history, without evidence of diabetes, hypertension, and smoking. Her blood count, and renal and liver functions were normal. She has been taking substitution therapy for hypothyroidism. Echocardiography showed no wall motion abnormalities, cardiac enzymes were in the normal range, and the patient was referred to coronarography. Coronarography revealed angiographically mild‐to‐intermediate lesion of the distal left‐main (LM), as well as intermediate lesions of circumflex (Cx), obtuse marginal and right coronary artery (RCA) with good flow (Figure 1A‐C). We decided to use fractional flow reserve (FFR) to investigate hemodynamic significance of all lesions using Pressure wire X (Abbott Vascular, Abbott Park, Illinois, USA) and following intracoronary adenosine administration (Figure 1). FFR to LAD, Cx, and RCA was 0.88, 0.84 (Figure 1D), and 0.92 respectively, ruling out hemodynamic significance and allowing PCI deferral. However, due to clinical presentation as well as some angiographic ambiguities including luminal border haziness of the lesion in the LM, we decided to perform also optical coherence imaging (OCT). OCT pullback using Dragonfly Optis catheter (Abbott Vascular, Abbott Park, Illinois, USA) revealed minimal lumen area of 7.9 mm^2^ with area percent stenosis of 50.4% but with multiple imaging phenomena demonstrating plaque instability that include small endothelial rupture, erosions, minor white and red thrombi, large subluminal lipid pool with reduced cap thickness (35µm), and clustering of macrophages beneath the cap surface (Figure 1E‐F). Taking into account clinical presentation of unstable angina, and OCT findings identifying LM stenosis as the culprit lesion, we decided to perform PCI of LM. Ostial involvement of LAD and Cx was not evident by OCT imaging, favoring provisional stenting strategy for distal left‐main intervention. Direct provisional bifurcation stenting LM‐LAD was performed with drug‐eluting stent implantation sized according to OCT left‐main measurements showing average lumen‐to‐lumen distance of 3,7mm (Xience Sierra 3.5 × 24 mm; Abbott Vascular, Abbott Park), with further optimization of results using noncompliant balloons for distal (NC Trek 3.5 × 12 mm; Abbott Vascular, Abbott Park) and proximal main vessel (NC quantum 5 × 6 mm; Boston Scientific Way Marlborough) to correct distal underexpansion and proximal and ostial malapposition arising from left‐main tubular tapering. Final angiographic and OCT images showed optimal reconstruction of distal left‐main bifurcation anatomy (minimal stent area LM 15.6 mm^2^, and LAD 11.4 mm^2^) without residual stenosis, dissections and malapposition (Figure 2). The patient was discharged in good clinical condition on dual antiplatelet therapy (aspirin 100 mg, and clopidogrel 75 mg bid for 1 month followed by 75 mg), statins (rosuvastatin 20 mg), and beta‐blockers (bisoprolol 5 mg), without any chest pain during next 6 months of follow‐up period.

## 3 DISCUSSION

Since both FFR and OCT assess different but complementary features of coronary stenosis, it seems rational that in certain clinical and angiographic scenarios, both methods should be performed to determine need for the intervention. Consequently, in patients with acute coronary syndrome and a lesion in LM of ambiguous severity and angiographic appearance, FFR and OCT should not be competitive but complementary to gain best insight into all aspects of coronary lesion.

Previous data on FFR for evaluation of intermediate LM stenosis in patients with stable angina have demonstrated excellent outcome in patients with negative FFR and deferred PCI.^6^ Although stenosis assessment by FFR in the case of LM is more challenging in comparison with non‐LM stenosis due to the requirement for disengagement of the guiding catheter and an inability to administer intracoronary adenosine, FFR/iFR has been recommended as a standard of care to guide decision on intervention.^1^ In addition, newer angiography‐based indices as quantitative flow ratio of QFR, based on computational fluid dynamic analysis, also proved to be useful in discriminating functionally significant stenosis, with an excellent correlation with FFR values, and considerable potential advantages in terms of costs and safety for complex interventions involving LM.^7^ Regarding imaging techniques, OCT and IVUS have limited diagnostic accuracy for identification of hemodynamically significant lesion, except for LM coronary stenosis where high correlation between functional significance by FFR and anatomical severity by IVUS/OCT has been demonstrated,^8^ leading to recommended and frequent clinical application of imaging to guide PCI in LM.^1^

However, the role of FFR in patients with ACS without ST elevation is less well established.^9^ FAME trial ^10^ with 1/3 of ACS patients demonstrated similar outcome in ACS and stable patients, whereas FAMOUS‐NSTEMI ^11^ in 350 patients randomized to angiography vs. FFR‐guided revascularization has shown no difference in 12 months of outcome, but with 22% change in strategy in FFR arm and consequently lower rates of revascularizations. Although underpowered, both studies did not demonstrate unfavorable role of FFR in patients with ACS. Finally, Escaned et al^12^ evaluated clinical outcome of patients deferred from revascularization on the basis of iFR and FFR in stable angina and ACS from 4486 patients. Although deferral was more frequent in iFR group (45% vs 50%), both techniques resulted in very low rate of adverse events of around 4%. However, patients with ACS had slightly and significantly more adverse events compared with patients presented with stable angina after 1 year (5.9% vs 3.6%, *P* = .04).

Role of OCT in identifying lesions that are to be treated remains open due to frequent identification of adverse angiographic features beyond angiography alone, implicating high‐risk lesion. Prati et al ^13^ have demonstrated that patients undergoing PCI with OCT guidelines required in 35% further intervention that translated into significantly lower rate of cardiac death and myocardial infarction. In the only randomized study comparing OCT with FFR‐guided PCI ^14^ that includes 350 patients with 446 intermediate lesions, OCT guidance (including stenosis severity and plaque rupture as indication for PCI) was associated with lower rate of composite major adverse cardiac events or angina over 13 months (14.8% vs 8%). Recently, CLIMA study ^15^ in more than 1000 patients identified certain OCT high‐risk features—intimal cap thickness of less than 75 µm findings, stenosis severity, long lipid arch, and OCT‐defined macrophages—as predictors of future adverse hard clinical events, death and target lesion related myocardial infarction. These findings are consistent with some of the high‐risk features evident in our patient—plaque rupture/erosion combined with thin fibrous cap overlaying lipid pool with clusters of macrophages. These adverse OCT findings combined with a location of the lesion in the left‐main stem, together with unstable clinical presentation and absence of contraindications for intervention over‐ruled initial decision to comply with good FFR measurements and defer PCI, but to perform intervention.

Regarding nonintervention in patients with ACS, the EROSION trial ^16^ has found that majority of patients (92.5%) with ACS caused by plaque erosion managed with aspirin and ticagrelor without stenting remained free of major adverse cardiovascular event for ≤1 year, and suggested that the nonintervention management may be an alternative option in these patients. However, the study was not randomized and compared to intervention, majority of the patients had ST elevation, thrombectomy was performed in most of the cases, and none of the patients had LM lesion.^16^

## 4 CONCLUSIONS

Adequate treatment of intermediate LM with negative FFR in the presence of ongoing chest pain and myocardial ischemia remains challenging. Due to large myocardium at risk and potential detrimental prognosis, it seems prudent to perceive all quantitative and qualitative lesion characteristics in more details, even appearing angiographically mild. For the clinical settings of acute coronary syndrome without ST elevation, both FFR and OCT imaging seem to be reasonable approach for the interrogation of patients with LM of mild‐to‐intermediate and/or ambiguous stenosis severity without clear evidence of thrombosis. Nevertheless, since we do not know what would be the outcome of deferring intervention in mild‐to‐intermediate culprit and high‐risk LM lesion with negative FFR (“blindness of coronary physiology”), the answer on the outcome of aggressive medical therapy, and not intervention, remains elusive.

## CONFLICT OF INTEREST

None declared.

## AUTHOR CONTRIBUTIONS

AA‐N: involved in case performance, drafted the manuscript, critically evaluated the manuscript, and approved the final version of the manuscript. WA: involved in case performance, served as a professional expertise of the case, and critically evaluated the manuscript. ZM: served as a professional expertise of the case, drafted the manuscript, and critically evaluated the manuscript. BB: involved in case performance, wrote the manuscript, performed the final analysis, and approved the final version of the manuscript.

## ETHICAL APPROVAL

Treatment of the patients has been performed according to good clinical practice and patients’ informed consent related to clinical diagnostic and treatment procedures of our hospital.
